# Supplementary material for: Molecular basis of FIGNL1 in dissociating RAD51 from DNA and chromatin
Source: Science. Author manuscript; Available in PMC 2025 Jan 27. (PMC7617353; doi:10.1126/science.adr7920)
Supplement: Supplementary Materials [file EMS202702-supplement-Supplementary_Materials.pdf]

Science
AAAS

Supplementary Materials for

Molecular basis of FIGNL1 in dissociating RAD51 from DNA and chromatin

Alexander Carver[a,b,d], Tai-Yuan Yu[c,d], Luke A Yates[a,b], Travis White[c], Raymond Wang[c], Katie Lister[a,b], Maria Jasin[c,*], and Xiaodong Zhang[a,b,*]

[a]DNA Processing Machines Laboratory, Francis Crick Institute, London, UK
[b]Section of Structural and Synthetic Biology, Department of Infectious Disease, Imperial College London, UK
[c]Developmental Biology Program, Memorial Sloan Kettering Cancer Center, New York, USA
[d]These authors contributed equally to this study.

Corresponding authors: Xiaodong.zhang@imperial.ac.uk, jasinm@mskcc.org

The PDF file includes:

Materials and Methods
Figs. S1 to S17
Tables S1 to S2
References 57-72

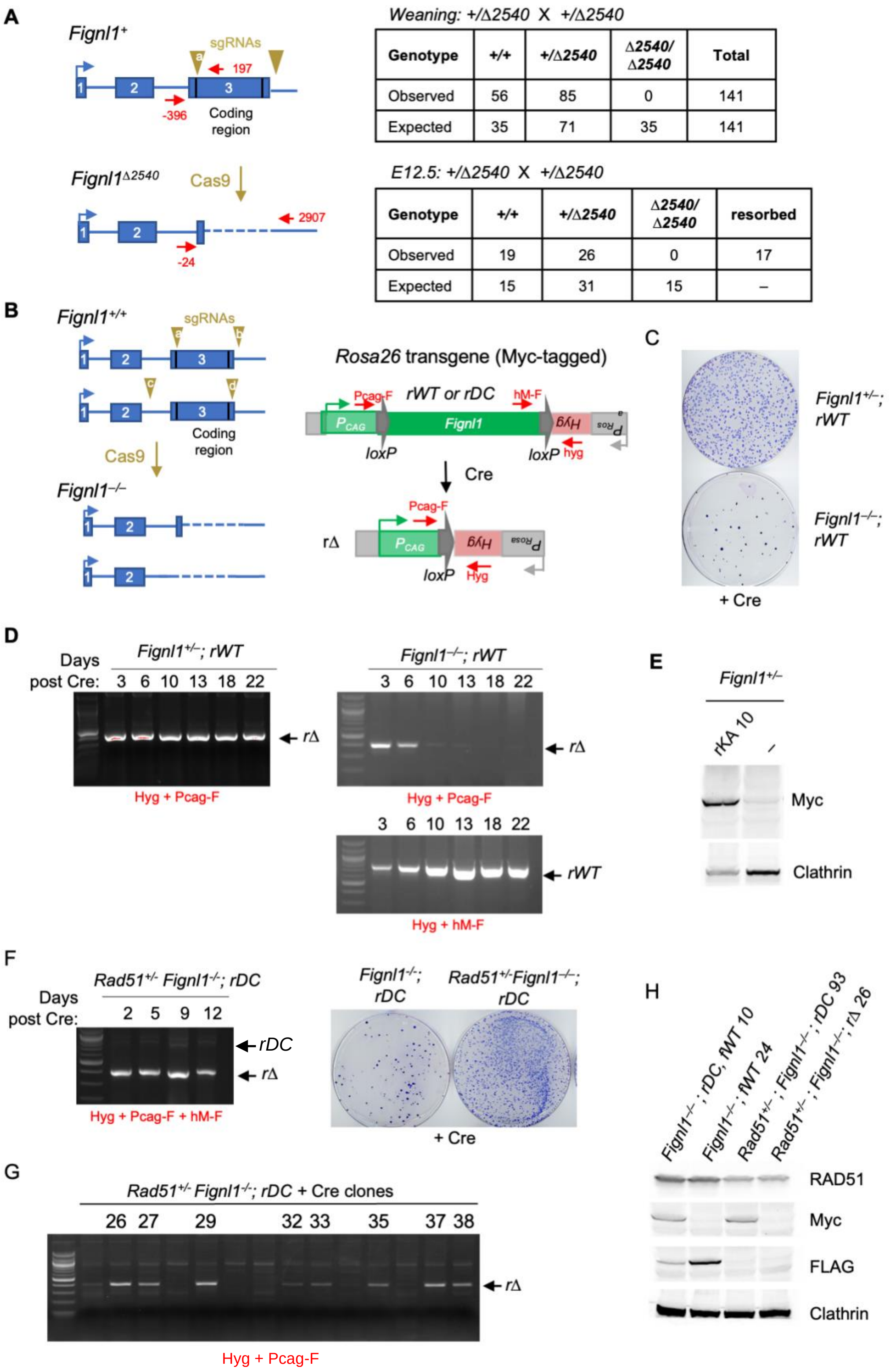

Weaning: +/Δ2540 X +/Δ2540

Genotype	+/+	+/Δ2540	Δ2540/ Δ2540	Total
Observed	56	85	0	141
Expected	35	71	35	141

E12.5: +/Δ2540 X +/Δ2540

Genotype	+/+	+/Δ2540	Δ2540/ Δ2540	resorbed
Observed	19	26	0	17
Expected	15	31	15	–

Fig. S1.

***Fignl1**$^{-/-}$* **mice and cells are not viable, but *Rad51* heterozygosity rescues cellular viability.**

(**A)** *Fignl1* mutant mice die prior to embryonic day 12.5. The *Fignl1*$^{\Delta 2540}$ allele was generated using CRISPR-Cas9 with two sgRNAs that delete the entire coding region. The position of the primers used for genotyping is shown relative to the ATG. No *Fignl1*$^{\Delta 2540/\Delta 2540}$ mutant mice were obtained either at weaning or at E12.5. (**B)** *Fignl1* conditional cell system. Wild-type *Fignl1* expressed from a P_{CAG} promoter and flanked by loxP sites was targeted to the *Rosa26* locus (*rWT*). Correctly targeted cells were selected using *Hyg* expression from the *Rosa26* promoter, generating *Fignl1*$^{+/+}$; *rWT* cells. The endogenous *Fignl1* alleles in the *Fignl1*$^{+/+}$; *rWT* cells are deleted in two steps, first with sgRNAs (a+b) to delete one allele, generating *Fignl1*$^{+/-}$*; rWT* cells, followed by sgRNAs (c+d) to delete the other allele, generating *Fignl1*$^{-/-}$*; rWT* cells. Cre expression was then used to delete the wild-type *Fignl1* transgene *rWT* to generate the *r*Δ allele. Primers used to amplify either *rWT* or *rΔ* are indicated. **(C)** Reduced colony formation of *Fignl1*$^{-/-}$ cells in comparison to *Fignl1*$^{+/-}$ cells upon deletion of *rWT* upon Cre expression. **(D)** Similarly, loss of *rWT* results in impaired proliferation of *Fignl1*$^{-/-}$ cells. Genomic DNA was collected at the indicated days post transfection of the Cre expression vector and amplified by primers shown in **B**. While deletion at the *Rosa26* locus (*r*Δ*)* was readily detected at days 3 and 6 post-Cre expression in *Fignl1*$^{-/-}$*; rWT* cells, it was not observed at later time points, indicating that *Fignl1*$^{-/-}$; *r*Δ cells were out competed over time by cells that had not deleted *rWT*. **(E)** Western blotting showing Myc-tagged FIGNL1 K456A (KA) expression in *Fignl1*$^{+/-}$*; rKA* cells used in **Fig. 1A. (F)** *Rad51* heterozygosity rescues cellular viability of *Fignl1* null mice. A *Fignl1* conditional allele was set up in *Rad51* heterozygous cells containing the *DC* allele at the *Rosa26* locus (*rDC*). Deletion of *rDC* was observed at late time points after Cre expression (9 and 12 days), indicating that *Fignl1*$^{-/-}$ cells are viable with *Rad51* heterozygosity. The high frequency of colony formation post Cre expression supports that *Rad51* $^{+/-}$ *Fignl1*$^{-/-}$ *;* *r*Δ cells are viable. **(G)** Individual *Rad51*$^{+/-}$ *Fignl1*$^{-/-}$; *r*Δ colonies were identified after Cre expression from plates as in **F**, with confirmed deletion of the entirety of FIGNL1. **(H)** Reduced RAD51 with *Rad51* heterozygosity and absent Myc-tagged FIGNL1 in a clone of *Rad51*$^{+/-}$ *Fignl1*$^{-/-}$ *;* *r*Δ cells.

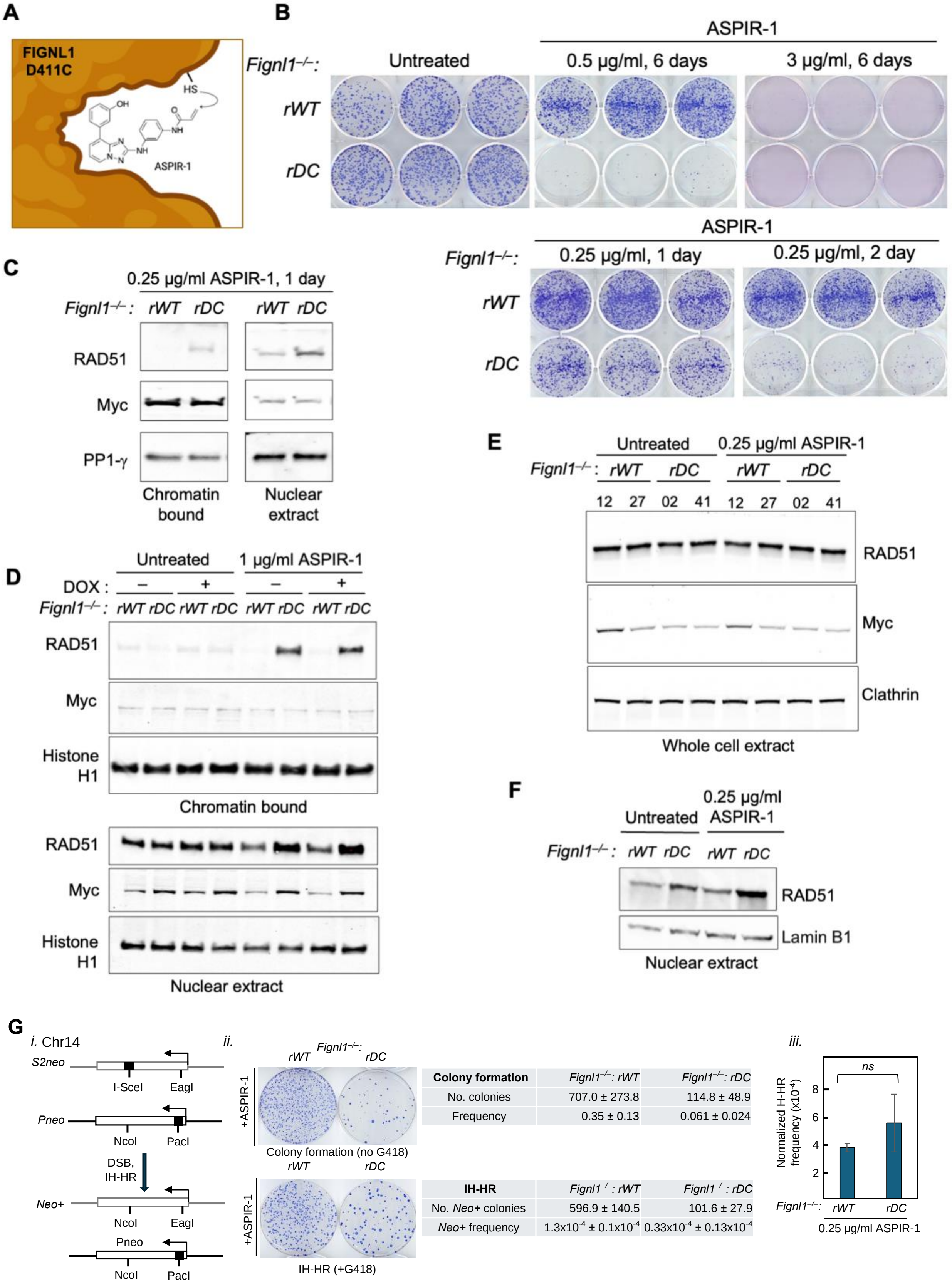
A
FIGNL1
D411C
HS
OH
NH
N
ASPIR-1
B
ASPIR-1
Fignl1−/−:
Untreated
0.5 µg/ml, 6 days
3 µg/ml, 6 days
rWT
rDC
ASPIR-1
Fignl1−/−:
0.25 µg/ml, 1 day
0.25 µg/ml, 2 day
rWT
rDC
C
0.25 µg/ml ASPIR-1, 1 day
Fignl1−/− : rWT rDC rWT rDC
RAD51
Myc
PP1-γ
Chromatin bound
Nuclear extract
D
Untreated
1 µg/ml ASPIR-1
DOX : – + – +
Fignl1−/− : rWT rDC rWT rDC rWT rDC rWT rDC
RAD51
Myc
Histone H1
Chromatin bound
RAD51
Myc
Histone H1
Nuclear extract
E
Untreated
0.25 µg/ml ASPIR-1
Fignl1−/− : rWT rDC rWT rDC
12 27 02 41 12 27 02 41
RAD51
Myc
Clathrin
Whole cell extract
F
Untreated
0.25 µg/ml ASPIR-1
Fignl1−/− : rWT rDC rWT rDC
RAD51
Lamin B1
Nuclear extract
G
i. Chr14
S2neo
I-SceI
EagI
Pneo
NcoI
PacI
DSB, IH-HR
Neo+
NcoI
EagI
Pneo
NcoI
PacI
ii.
Fignl1−/−:
rWT
rDC
+ASPIR-1
Colony formation (no G418)
Colony formation | Fignl1−/−: rWT | Fignl1−/−: rDC
No. colonies | 707.0 ± 273.8 | 114.8 ± 48.9
Frequency | 0.35 ± 0.13 | 0.061 ± 0.024
rWT
rDC
+ASPIR-1
IH-HR (+G418)
IH-HR | Fignl1−/−: rWT | Fignl1−/−: rDC
No. Neo+ colonies | 596.9 ± 140.5 | 101.6 ± 27.9
Neo+ frequency | 1.3x10-4 ± 0.1x10-4 | 0.33x10-4 ± 0.13x10-4
iii.
ns
Normalized H-HR frequency (x10-4)
8
6
4
2
Fignl1−/−: rWT rDC
0.25 µg/ml ASPIR-1

Fig. S2. Chemical inhibition of FIGNL1 ATPase domain leads to cell lethality and is associated with RAD51 chromatin accumulation. (**A**) Chemical inhibition of mouse FIGNL1 D411C with ASPIR-1. ASPIR-1 is N-(3-((8-(3-hydroxyphenyl)-[1,2,4]triazolo[1,5-a]pyridin-2-yl)amino)phenyl)acrylamide (*18*). The sulfhydryl group of the introduced in cysteine in the FIGNL1 catalytic site forms a covalent linkage with the acrylamide electrophile in ASPIR-1. ATPase activity of the cognate human FIGNL1 mutant D402C is reduced in a dose-dependent manner by ASPIR-1; in the absence of ASPIR-1, the D402C mutant has a 2-fold reduction in catalytic activity. The drawing was created with BioRender software. (**B**) Titration of ASPIR-1 in *Fignl1$^{-/-}$; rWT* and *Fignl1$^{-/-}$; rDC* cells. Continuous ASPIR-1 exposure at 0.5 µg/ml leads to lethality only of *Fignl1$^{-/-}$; rDC* cells, whereas 3 µg/ml is also lethal to control cells. One day exposure to 0.25 µg/ml ASPIR-1 is sub-lethal, whereas 2-day exposure kills most *Fignl1$^{-/-}$; rDC* cells. (**C**) A sublethal dose of ASPIR-1 is sufficient to lead to RAD51 accumulation in the chromatin fraction of *Fignl1$^{-/-}$; rDC* cells. Here and below the *rWT* and *rDC* alleles encode Myc-tagged FIGNL1. (**D**) Similar to C with additional conditions. Cells were precultured for 3 days with doxycycline (1 µg/ml) where indicated to suppress BLM expression (*16*). Western blot analysis of chromatin or nuclear fractions demonstrates similar level of RAD51 chromatin accumulation with or without BLM. (**E**) Total RAD51 levels are unaffected by ASPIR-1 treatment. The same blot of whole cell extracts as in Fig. 1c with additional clones. (**F**) Nuclear extract from cellular fractionation from Fig. 1C is shown. Three independent clones 02, 10, 41 of *Fignl1$^{-/-}$; rDC* accumulated chromatin bound RAD51 in C, D, and F, respectively, in the presence of ASPIR-1. (**G**) ASPIR-1 treatment of *Fignl1$^{-/-}$*; *rDC* cells impairs survival but not homologous recombination. *i.* Inter-homolog homologous recombination (IH-HR) reporter in mouse ES cells. The *S2neo* gene contains an I-SceI site at the position of the NcoI site in the wild-type gene, and the *Pneo* gene contains an insertion of a PacI site at what is normally an EagI site. A DSB generated in *S2neo* by I-SceI endonuclease can lead to repair by IH-HR from *Pneo* resulting in *Neo*+ recombinants which are resistant to the drug G418. *ii.* Colony formation of *Fignl1$^{-/-}$*; *rDC* cells treated with 0.25 µg/ml ASPIR-1 for 1 day in the absence of G418 is reduced due to toxicity of ATPase inhibition. *Neo*+ colony formation is similarly reduced compared with *Fignl1$^{-/-}$; rWT* cells. *iii.* IH-HR frequencies were normalized within each experiment by dividing the *Neo*+ frequency with colony formation frequency (3 independent experiments).

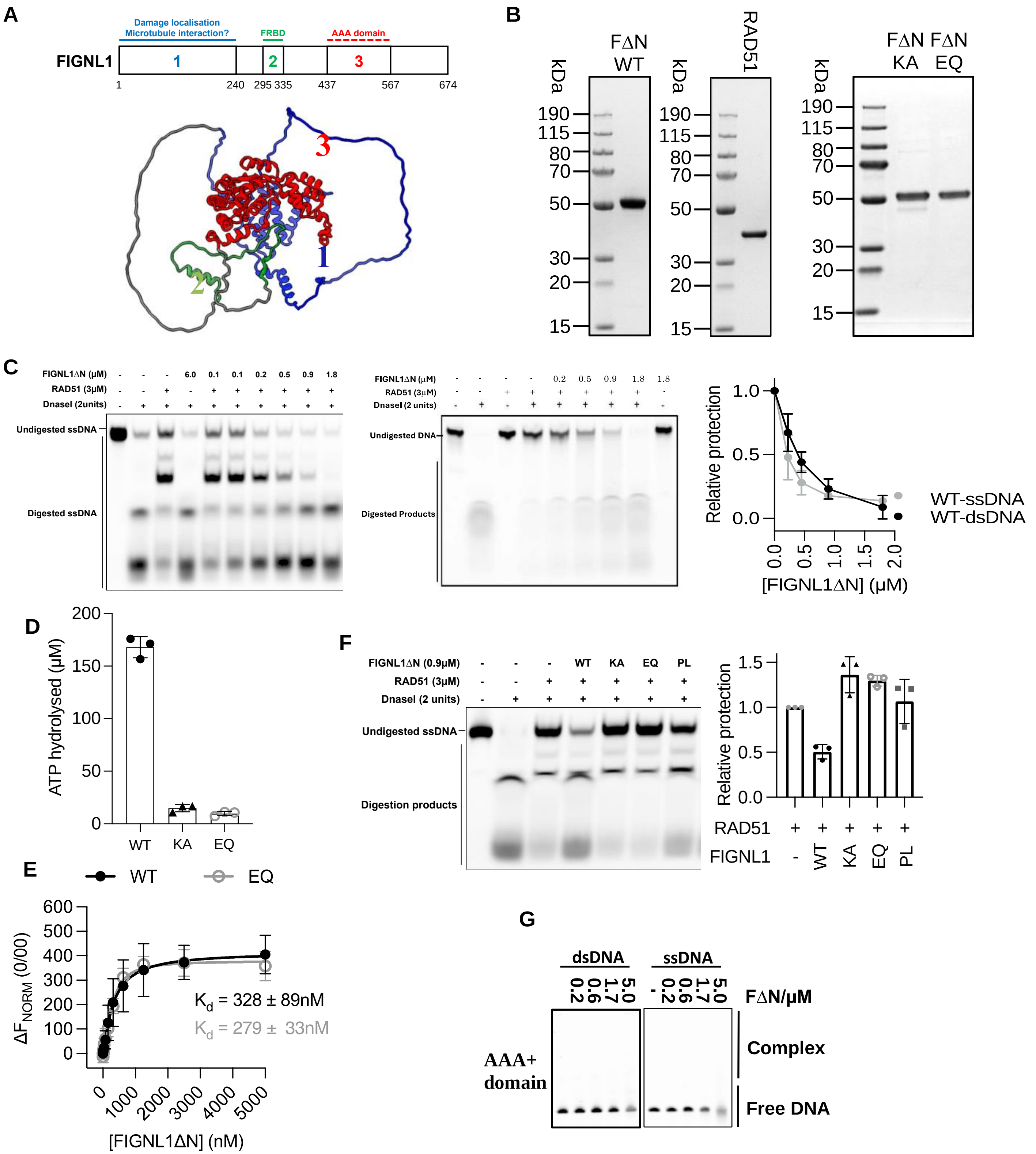

Fig. S3. In vitro characterization of FIGNL1ΔN and mutants.

(A) Domain architecture of FIGNL1 and AlphaFold2 model of full-length FIGNL1. (B) Images of gel showing quality of purified FIGNL1ΔN, RAD51, K447A and E501Q FIGNL1ΔN. (C) Image of gel showing nuclease protection of 60nt ssDNA and dsDNA by RAD51 in the presence of increasing concentrations of FIGNL1ΔN. right panel: Quantification of the undigested DNA. Each data point represents the mean ± s.d., n=3 for dsDNA and n = 2 for ssDNA. (D) Quantification of ATPase activity of E501Q, K447A and WT FIGNL1ΔN, n = 3, each data point represents the mean ± s.d. (E) Quantification of the interactions between WT and E501Q FIGNL1ΔN with RAD51 by MST, n = 3, each data point represents the mean ± s.d. (F) Image of gel showing nuclease protection of 60nt ssDNA by RAD51 in the presence of 0.9μM WT, Walker A – K447A, Walker B – E501Q and PL mutant of FIGNL1ΔN, and quantification of those nuclease protection assays. Each data point represents the mean ± s.d., n = 3.(G) EMSA data showing that the AAA+ domain of FIGNL1 does not bind to dsDNA or ssDNA.

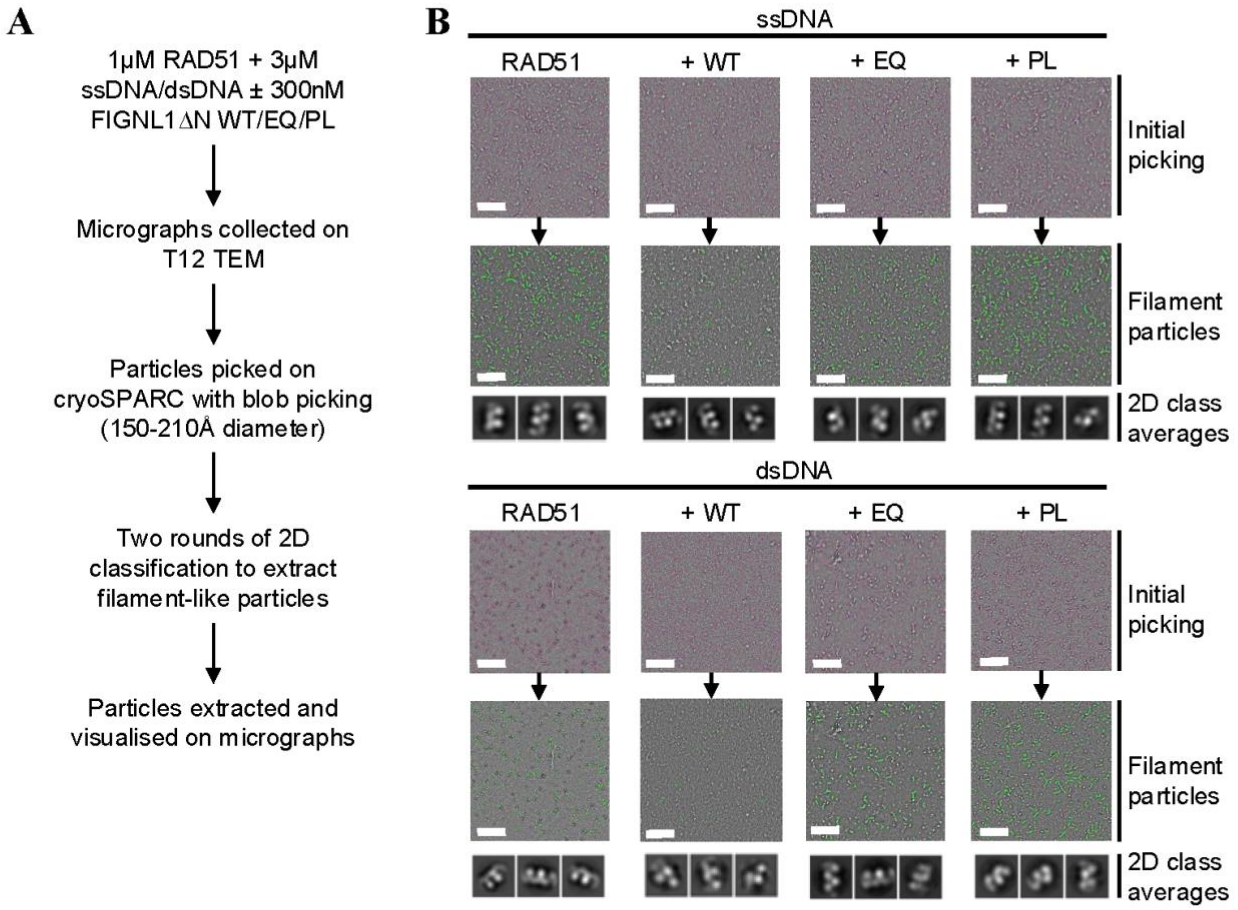

Fig. S4. RAD51 filament quantification.
(A). Processing pipeline of sample preparation, data collection and number of RAD51 filament-like particles per micrograph per condition (RAD51-ssDNA/dsDNA filaments in the absence and presence of WT, E501Q and PL mutant FIGNL1ΔN). (B) Representative micrographs and 2D class averages from each condition imaged and analysed by cryoSPARC processing. Scale bar = 200nm.

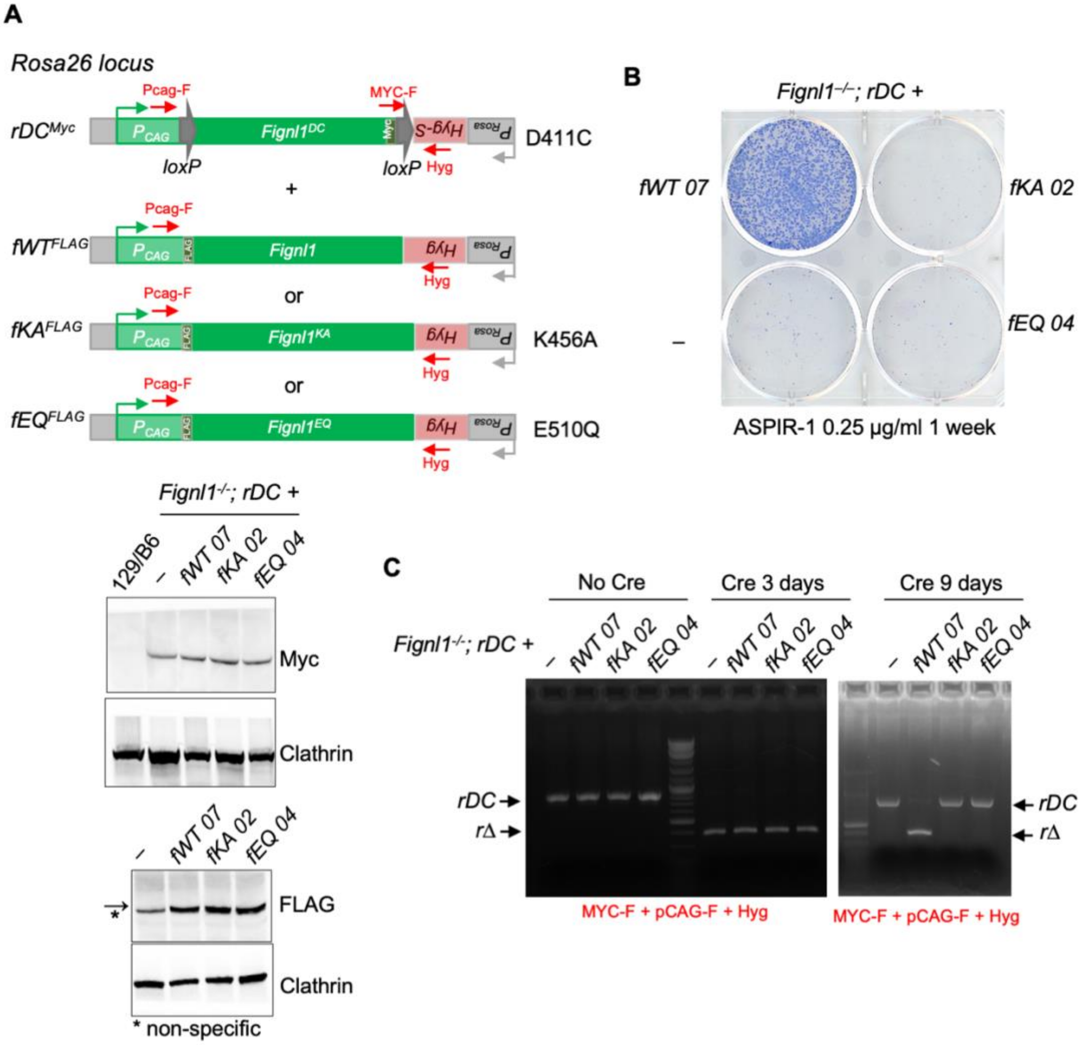

Fig. S5. FIGNL1 Walker A and Walker B mutants are not able to suppress cellular lethality upon loss or chemical inhibition of the *rDC* allele, as seen using a dual *Rosa26* expression system. (**A**) The dual system is set up in three steps. 1. Integration of the conditional Myc-tagged *rDC* allele in one *Rosa26* allele, 2. disruption of the *Hyg* marker (*Hyg-S*, or sensitive), 3. introduction of a series for FLAG-tagged alleles at the other *Rosa26* allele. Western blotting confirms expression of the alleles. (**B**) *Fignl1*$^{-/-}$*; rDC*^{Myc}*, fWT*^{FLAG} clones were resistant to ASPIR-1, indicating that the inhibited DC protein does not interfere with functional FIGNL1 protein. However, *Fignl1*$^{-/-}$*; rDC*^{Myc}*, fKA*^{FLAG} or *Fignl1*$^{-/-}$*; rDC*^{Myc}*, fEQ*^{FLAG} clones do not survive ASPIR-1 treatment, indicating that the Walker A and Walker B mutants, respectively, are unable (non-functional) to promote survival when the DC protein is inhibited. (**C**) Deletion of the *rDC* allele (*r*Δ) is stable in cells expressing wild-type FIGNL1 but not in cells expressing the Walker A and Walker B mutants, indicating that the *fKA* or *fEQ* expression impairs proliferation of cells. Genomic DNA was collected at the indicated days post transfection of the Cre expression vector and amplified by primers as shown in A.

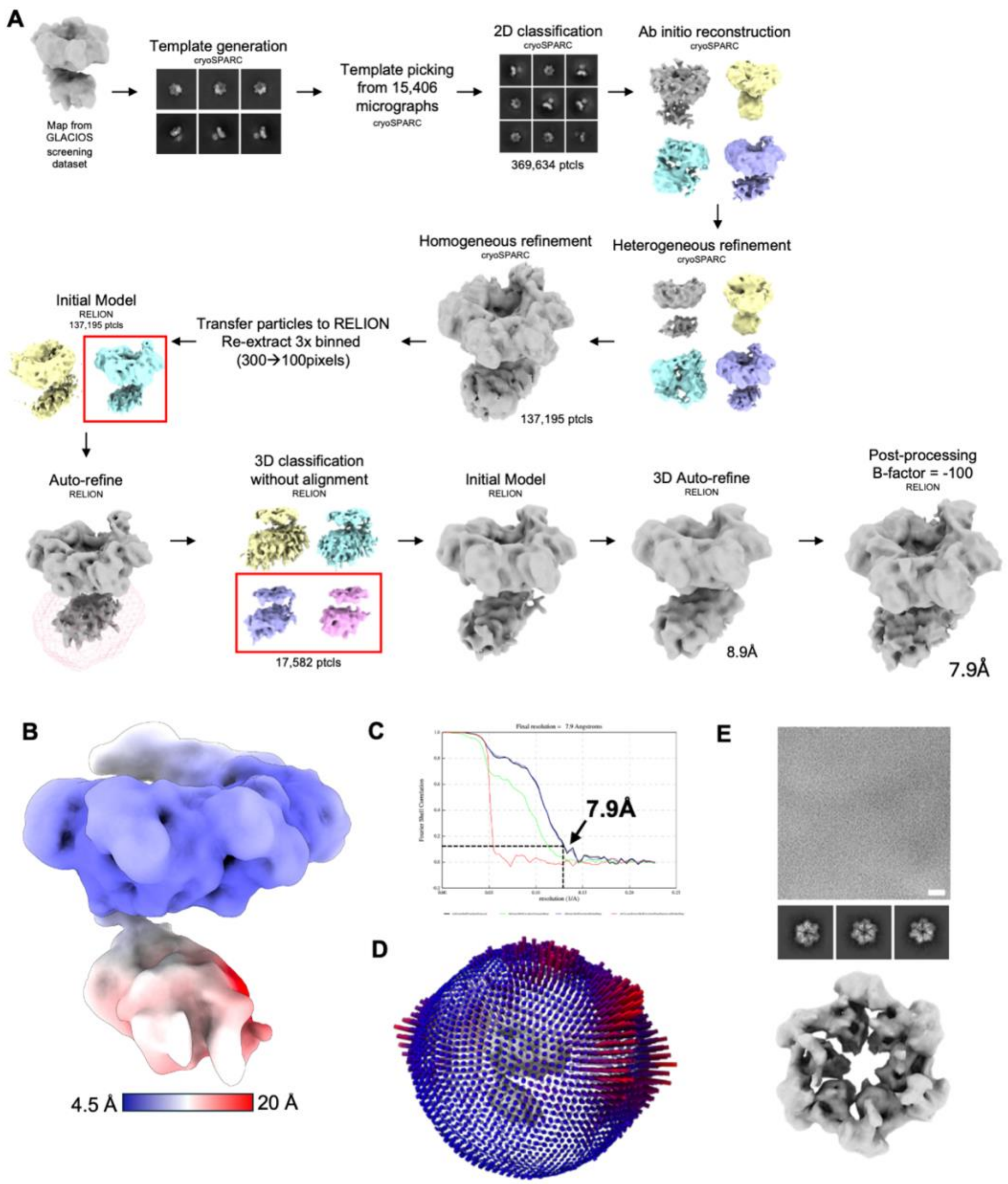

Fig. S6. Single-particle data analysis flowchart for the FIGNL1ΔN^{E501Q}-RAD51-ATP.Mg^{2+} complex. (**A**) Summary of the data processing pathway including the 2D classes, 3D model generation, 3D model selection, 3D classification and 3D refinement, resulting in the final reconstruction at 7.7 Å global resolution. (**B**) Local resolution distributions for the FIGNL1ΔN^{E501Q}-RAD51 cryoEM reconstruction. (**C**) Fourier shell correlation (FSC) plots. (**D**) Angular distribution of particles contributed to the final reconstruction. (**E**) Micrograph, 2D classes and 3D reconstruction of WT FIGNL1ΔN in the presence of ATPγS, which shows that it can form hexamers. FIGNL1ΔN was applied to lacey carbon copper grids with an ultrathin carbon layer and imaged using on a KRIOS TEM. Particles were picked, 2D classified, refined and post-processed to produce a map of ~10Å resolution. Scale bar = 50nm.

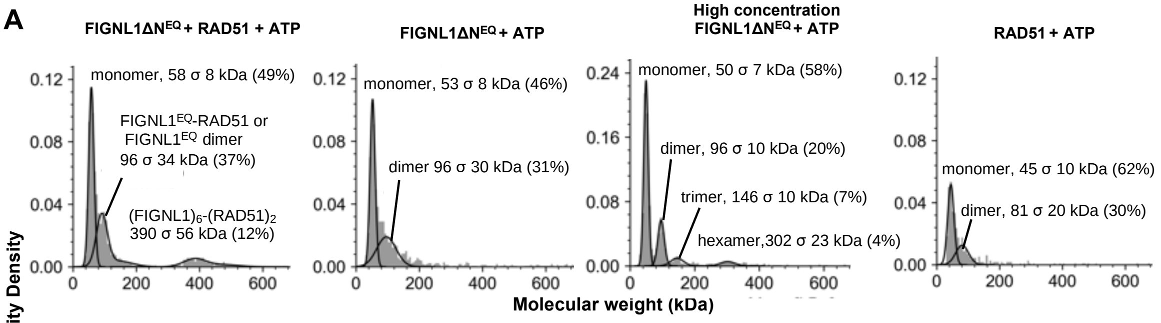

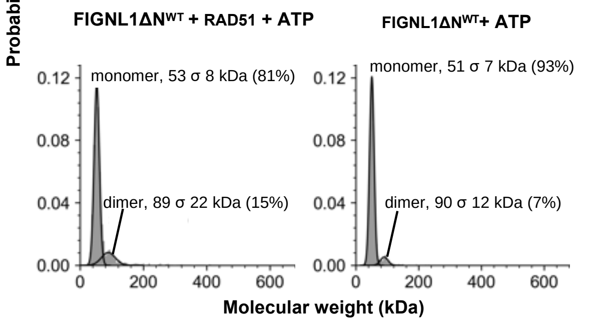

Protein	MW_{calc} (kDa)	MW_{obs} (kDa)
RAD51	36.96	45 σ 10
(RAD51) dimer	73.92	81 σ 20
FIGNL1ΔN$^{WT/EQ}$	46.02	51 σ 7 / 53 σ 8
(FIGNL1ΔN$^{WT/EQ}$) dimer	92.04	90 σ 12<br>96 σ 30
FIGNL1ΔN$^{WT/EQ}$-RAD51	82.98	
FIGNL1ΔN$^{WT/EQ}$ Hexamer	276.12	302 σ 23
(FIGNL1ΔN)$_6$-(RAD51)$_2$	350.06	390 σ 56

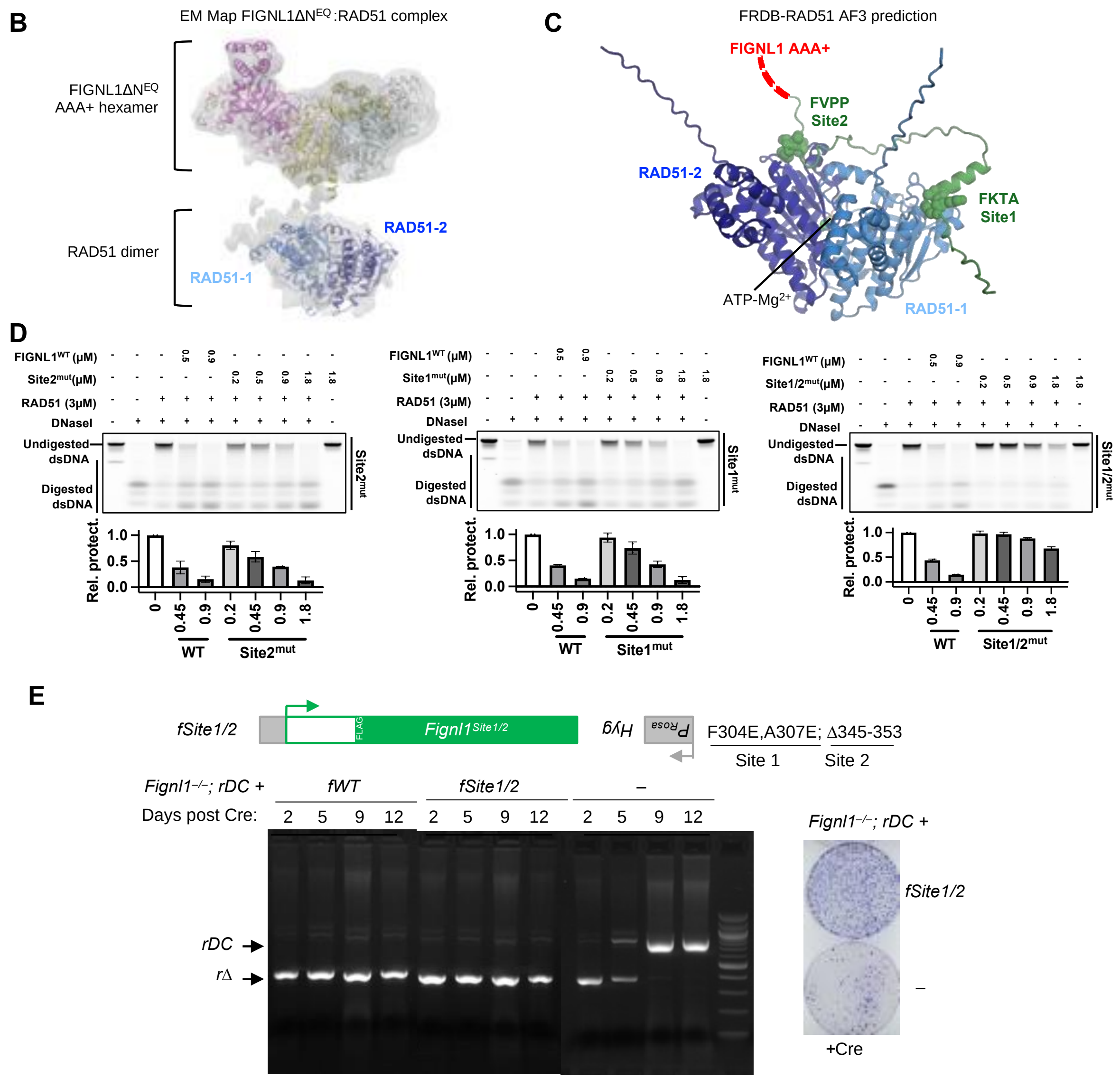

Fig. S7. Probing the interactions between FIGNL1 and RAD51.
(A) Mass distribution of FIGNL1ΔN (WT and EQ mutant), RAD51, and FIGNL1ΔN-RAD51 complexes measured by mass photometry in the presence of 0.8 mM ATP-Mg^{2+}. Observed masses (kDa) are indicated and the percentage of particles contributing to each peak is shown in brackets. Expected and measured masses are tabulated. The observed mass is larger than expected due to the crosslinking procedure. (B) Fitted atomic co-ordinates of a FIGNL1ΔN hexamer with a dimer of RAD51 in the low-resolution EM density. (C) AlphaFold3 (AF3) model of FIGNL1 FRBD in complex with a RAD51 dimer. AF3 more consistently predicts the FRBD interaction with a RAD51 trimer, which mimics a filament. (D) Nuclease protection assay showing the effect of WT FIGNL1ΔN, Site1 deleted ($Site1^{mut}$), Site2 deleted ($Site2^{mut}$) and both sites deleted ($Site1/2^{mut}$) formed on 60mer dsDNA coated by RAD51. Quantification was shown underneath. Each data point represents the mean ± s.d. n = 3. (E) The FLAG-tagged Site1/2 FIGNL1 mutant F304E, A307E, Δ345-353 mutant was expressed from the second allele in the dual *Rosa26* system (*Fignl1*$^{-/-}$*; rDC, fSite1/2*). Deletion of the *rDC* allele is detected at days 9 and 12 post-Cre transfection in *Fignl1*$^{-/-}$*; rDC fSite1/2* or *fWT* cells, while it is only initially observed in *Fignl1*$^{-/-}$*; rDC* cells and then is gone by the days 9 and 12, indicating that the Site1/2 mutant is still functional for supporting cells viability. This is supported by colony formation shown in the right panel. *Fignl1*$^{-/-}$*; r*Δ cells were unable to form colonies, whereas *Fignl1*$^{-/-}$*; r*Δ*, fSite1/2*^{FLAG} cells robustly formed colonies.

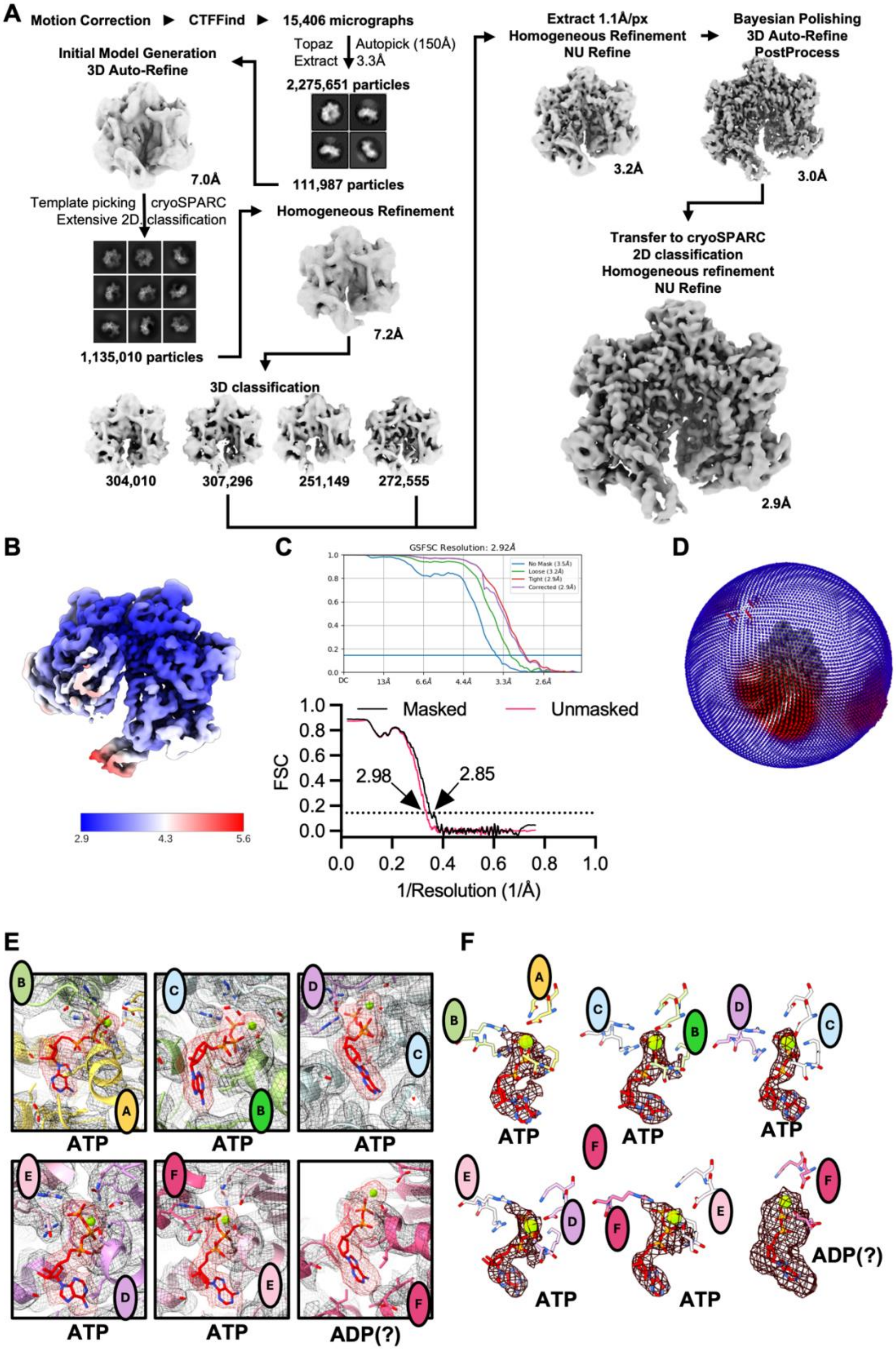
A
Motion Correction ► CTFFind ► 15,406 micrographs
Topaz
Extract
Autopick (150Å)
3.3Å
2,275,651 particles
Initial Model Generation
3D Auto-Refine
7.0Å
111,987 particles
Template picking
Extensive 2D
cryoSPARC
classification
1,135,010 particles
Homogeneous Refinement
7.2Å
3D classification
304,010
307,296
251,149
272,555
Extract 1.1Å/px
Homogeneous Refinement
NU Refine
3.2Å
Bayesian Polishing
3D Auto-Refine
PostProcess
3.0Å
Transfer to cryoSPARC
2D classification
Homogeneous refinement
NU Refine
2.9Å
B
2.9
4.3
5.6
C
GSFSC Resolution: 2.92Å
No Mask (3.5Å)
Loose (3.2Å)
Tight (2.9Å)
Corrected (2.9Å)
Masked
Unmasked
FSC
2.98
2.85
1/Resolution (1/Å)
D
E
ATP
ATP
ATP
ATP
ATP
ADP(?)
F
ATP
ATP
ATP
ATP
ATP
ADP(?)

Fig. S8. Single-particle analysis (SPA) of the FIGNL1ΔN^{E501Q}-RAD51 complex. (**A**) Data processing pipeline showing the pathway from raw movies to the final 2.9Å reconstruction of the FIGNL1ΔN hexamer bound to RAD51, focusing on FIGNL1 AAA+ domain. Classes taken to subsequent processing steps are outlined in red. (**B**) Local resolution estimates for the FIGNL1ΔN^{E501Q}-RAD51 cryoEM map. (**C**) Fourier shell correlation (FSC) plot and map-model FSC plot calculated in Phenix. (**D**) Angular distribution plot. (**E**) electron density and models surrounding each nucleotide binding pocket. (**F**) Density for nucleotide in each nucleotide binding pocket.

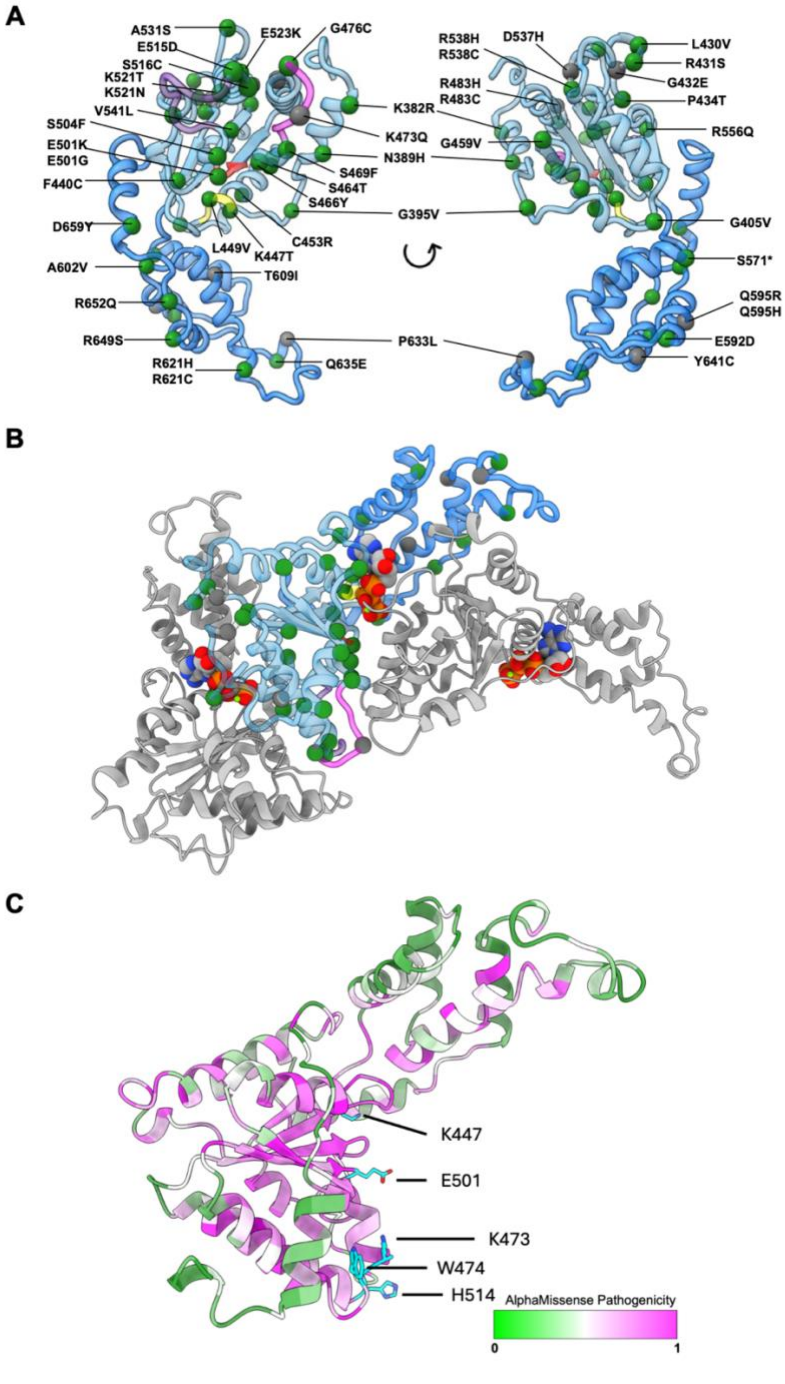

Fig. S9. Mutations found in patients with cancer or various of unknown significance (VUS) in other genetic disorders in the FIGNL1 AAA+ domain. VUS are shown in gray, cancer mutations in green, pore loop 1 in magenta, pore loop 2 in purple, Walker A motif in yellow and Walker B in red. (**A**) Specific mutations mapped onto monomers (**B**) mutations in the context of oligomers. (**C**) Pathogenicity scores were calculated with AlphaMissense(39). Scores from 0 (low pathogenicity) to 1 (high pathogenicity) are colored on the AlphaFold model of the FIGNL1 AAA+ domain.

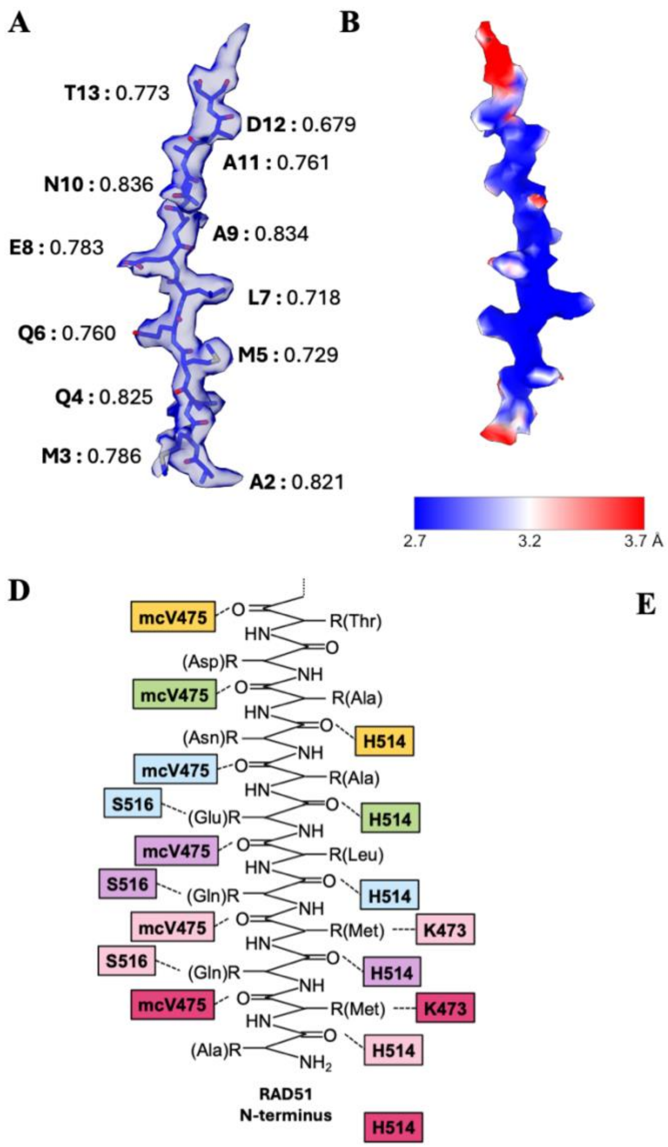

Fig. S10. The pore loops of FIGNL1 form a close network of interactions with the RAD51 N-terminus. (**A**) Calculated Q-scores for the N-terminus of RAD51 fitted into the peptide density from the 2.9Å reconstruction of the peptide-bound FIGNL1 AAA hexamer (22). (**B**) Local resolution estimates of the N-terminus of RAD51 as calculated in ResMap indicating high resolution of the N-terminus (**C**) The lysine and tryptophan residues of pore loop 1 of FIGNL1 intercalate the N-terminal peptide of RAD51. H514 of pore loop 2 potentially form hydrogen binding interactions with RAD51 N-terminal residues. (**D**) Schematic of putative H-bonds between pore loops 1 and 2 with the RAD51 N-terminus, main chain interactions are preceded by 'mc'. (**E**) Schematic of the interaction network created by the lysine-tryptophan dipeptide of pore loop 1 and H514 of pore loop 2. Orange dashed lines represent π-cation interactions. Red circles represent hydrophobic interactions from W474 to the hydrophobic side chains of RAD51.

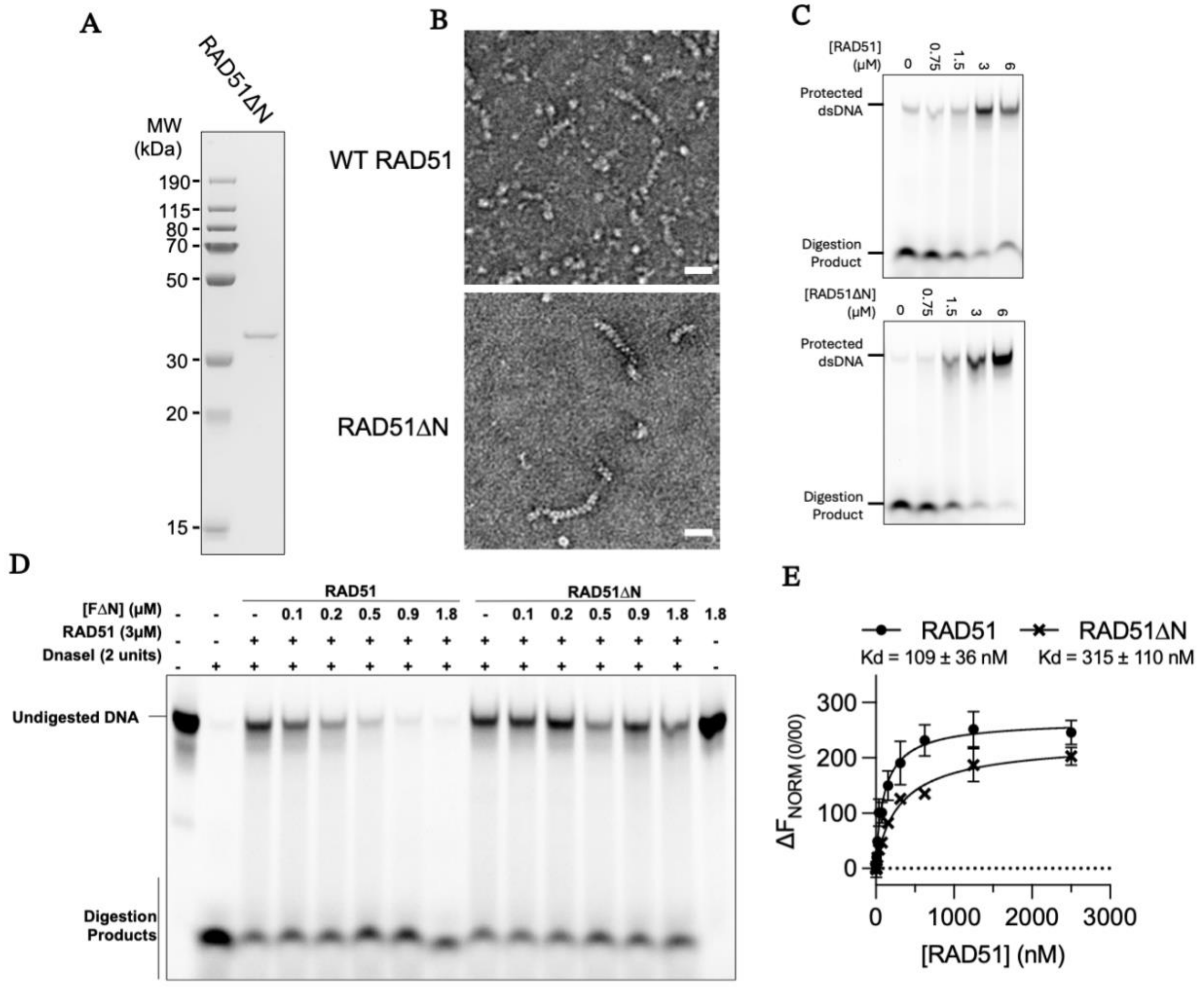

Fig. S11. Characterization of RAD51ΔN. (**A**) Image of gel showing quality of purified RAD51ΔN. (**B**) Representative micrographs of RAD51 and RAD51ΔN in complex with dsDNA. (**C**) Image of gel of nuclease protection of dsDNA by increasing concentrations of RAD51 and RAD51ΔN. (**D**) Image of gel of nuclease protection by RAD51 and RAD51ΔN in the presence of increasing concentrations of WT FIGNL1ΔN. (**E**) Quantification of interaction between labelled WT FIGNL1ΔN and WT RAD51 and RAD51ΔN as measured by microscale thermophoresis of AlexaFluor-647-labelled WT FIGNL1ΔN. Each datapoint represents the mean ± s.d., n = 3.

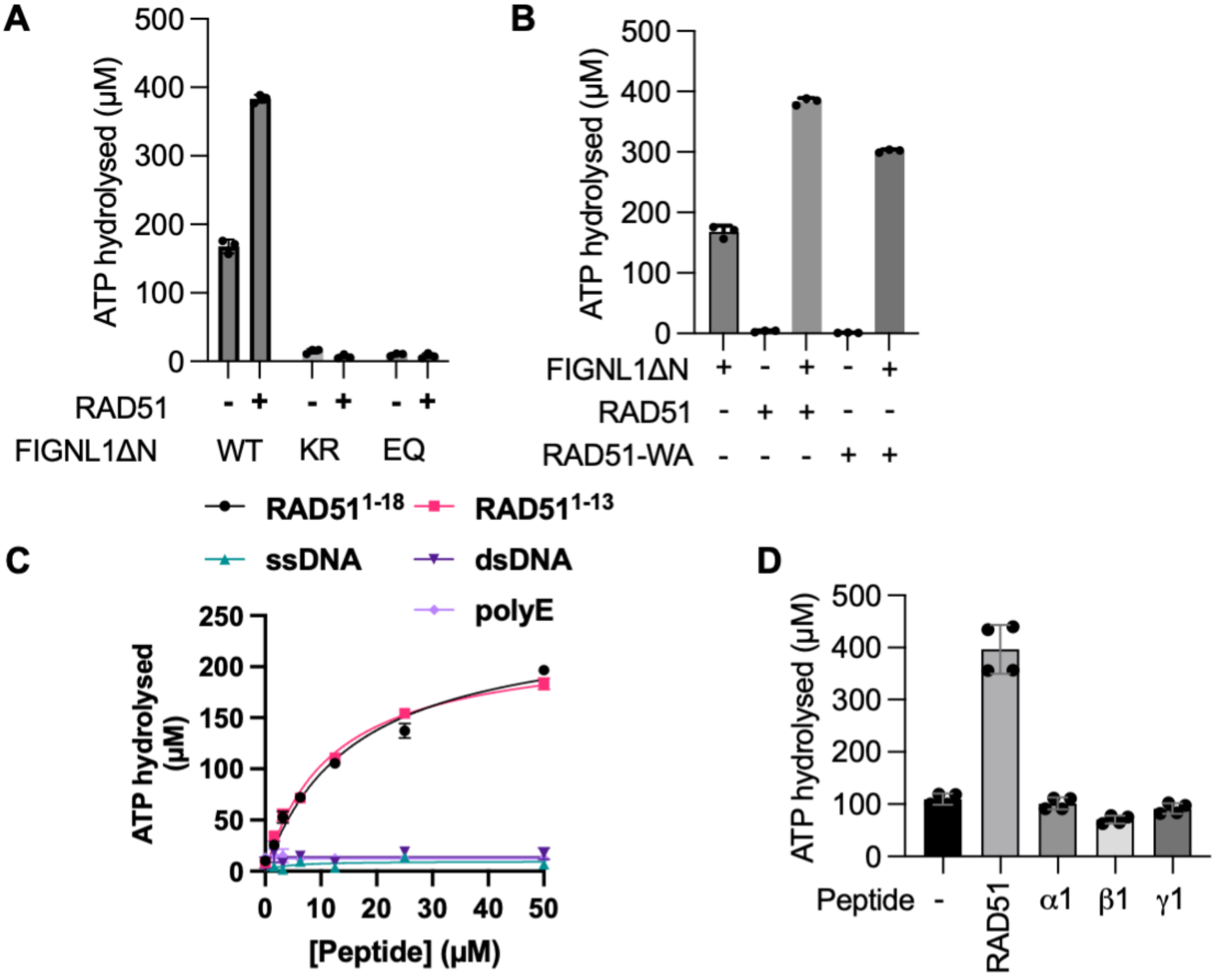

Fig. S12. Investigation of the effect of RAD51 on FIGNL1 ATPase activity. (**A**) ATP hydrolysis rate of FIGNL1DN, Walker A (K477R), Walker B (E501Q) mutant in the absence and presence of RAD51. (**B**) ATP hydrolysis rate of FIGNL1ΔN in the absence and presence of WT RAD51 and RAD51 Walker A mutant (RAD51-WA, K133R). (A) and (B) together support that RAD51 stimulates FIGNL1 activities while FIGNL1 does not stimulate RAD51 ATPase activity. (**C**) ATP hydrolysis by FIGNL1DN in the presence of mutated RAD51 N-terminal peptides (1-18 and 1-13), 60mer ssDNA and dsDNA and polyglutamate peptide. Each data point represents 3 independent measurements. (**D**) ATP hydrolysis of FIGNL1ΔN (1 mM) in the presence and absence of 50µM RAD51 N-terminal peptide and α, β and γ tubulin C-terminal peptides. Each data point represents the mean ± s.d.

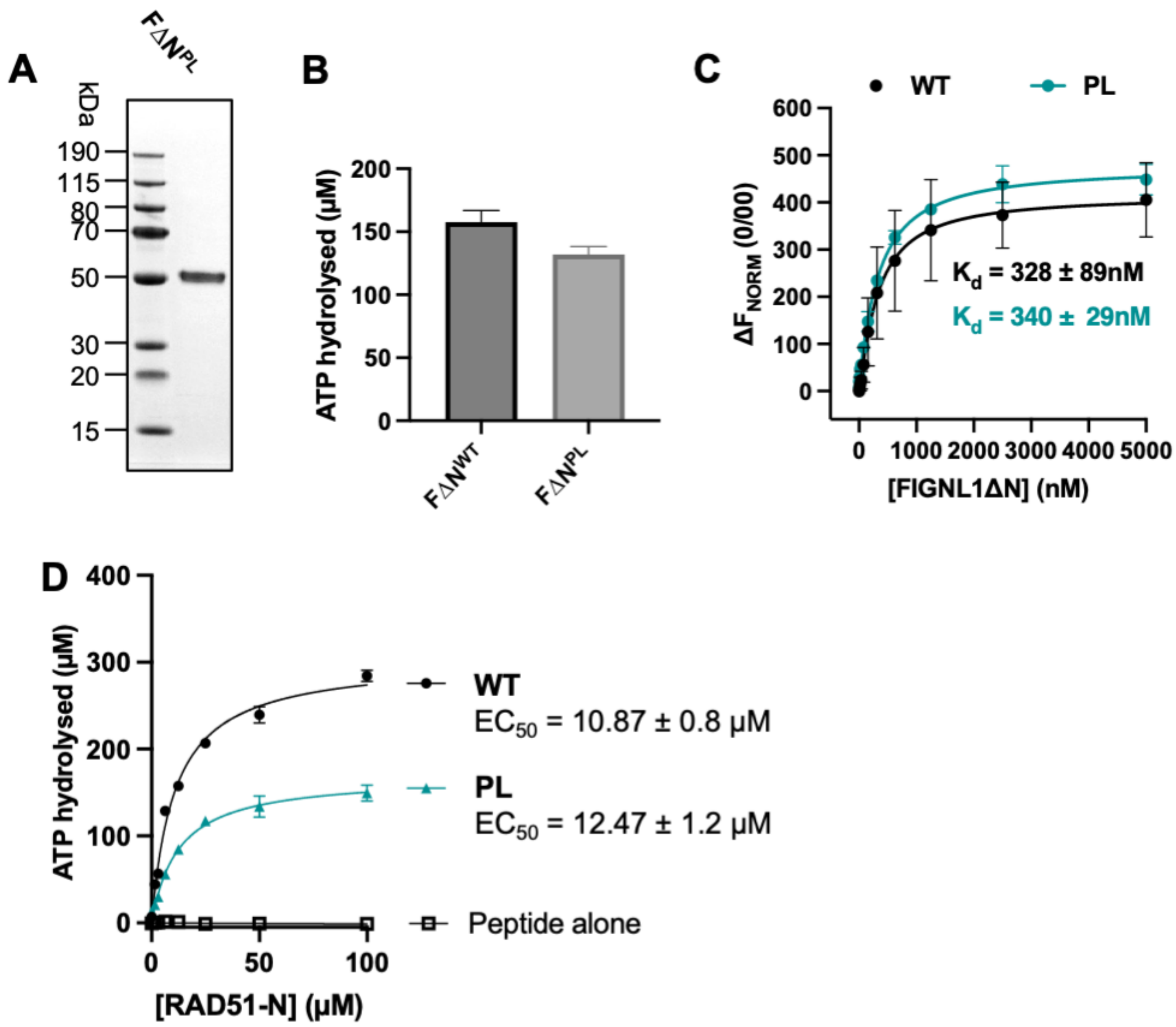

Fig. S13. Characterization of the PL mutant of FIGNL1ΔN and investigating the translocase activities of FIGNL1. (**A**) Image of gel showing quality of purified PL mutant FIGNL1ΔN (K473E, W474A). (**B**) Quantification of the ATPase activities of WT and PL FIGNL1ΔN, n = 3, data points represent the mean ± s.d. (**C**) Quantification of the WT and PL FIGNL1ΔN interaction with RAD51 as measured by microscale thermophoresis, n = 3, data points represent the mean ± s.d. WT data shown for comparison but it is the same data as shown in **Fig. S3E**. (**D**) Quantification of the ATPase activity of 100nM WT and PL FIGNL1ΔN in the presence of increasing concentrations of RAD51 N-terminal (RAD51-N) peptide. Each data point represents the mean ± s.d. ATPase activity of the PL mutant can be stimulated by RAD51.

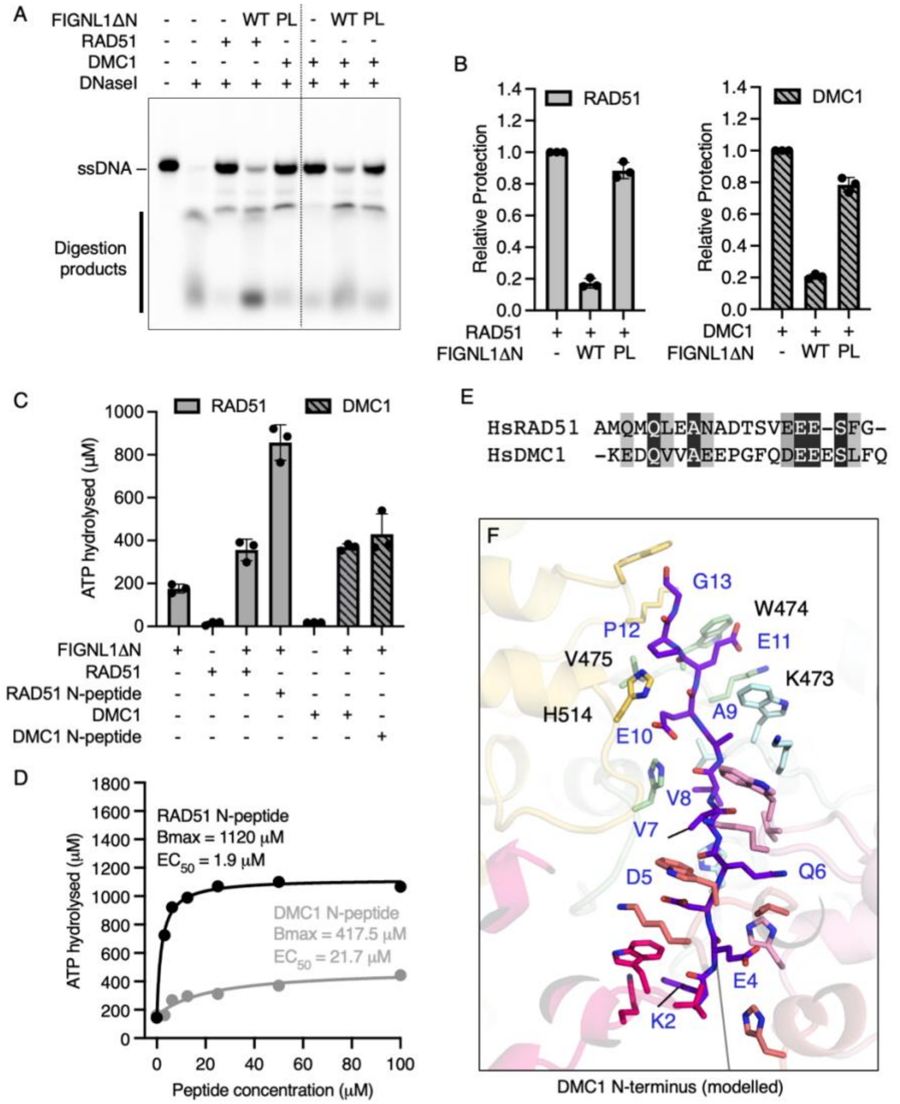

Fig. S14. FIGNL1ΔN acts upon DMC1. (**A**) Image of polyacrylamide gel showing nuclease protection of 60nt ssDNA by RAD51 or DMC1 alone and in the presence of 0.9μM WT or PL (pore loop) FIGNL1ΔN. (**B**) Quantification of replicated nuclease protection experiments. Bar represents mean ± standard deviation. Individual data points for each replicate is shown. Relative protection is normalized to either RAD51 or DMC1 alone for the respective experiments. (**C**) ATP hydrolyzed by FIGNL1ΔN (1μM) in the absence and presence of 1μM RAD51, 50μM RAD51 N-terminal peptide, 1μM DMC1, or 50μM DMC1 N-terminal peptide. Bar represents mean ± standard deviation. Individual data points for each replicate is shown. (**D**) Stimulation of FIGNL1DN ATPase activity by RAD51 and DMC1 N-terminal peptides at 1μM FIGNL1. (**E**) Amino acid sequence alignment between the N-terminal regions of human RAD51 and DMC1. Shaded residues are similar (grey) or identical (black). (**F**) Modelling of the DMC1 N-terminus into the pore of FIGNL1, based upon the FIGNL1 hexamer-RAD51 cryoEM complex determined in this study, suggests it can be accommodated. Pore loop side chains are shown as sticks and subunits colored as for Fig 2.

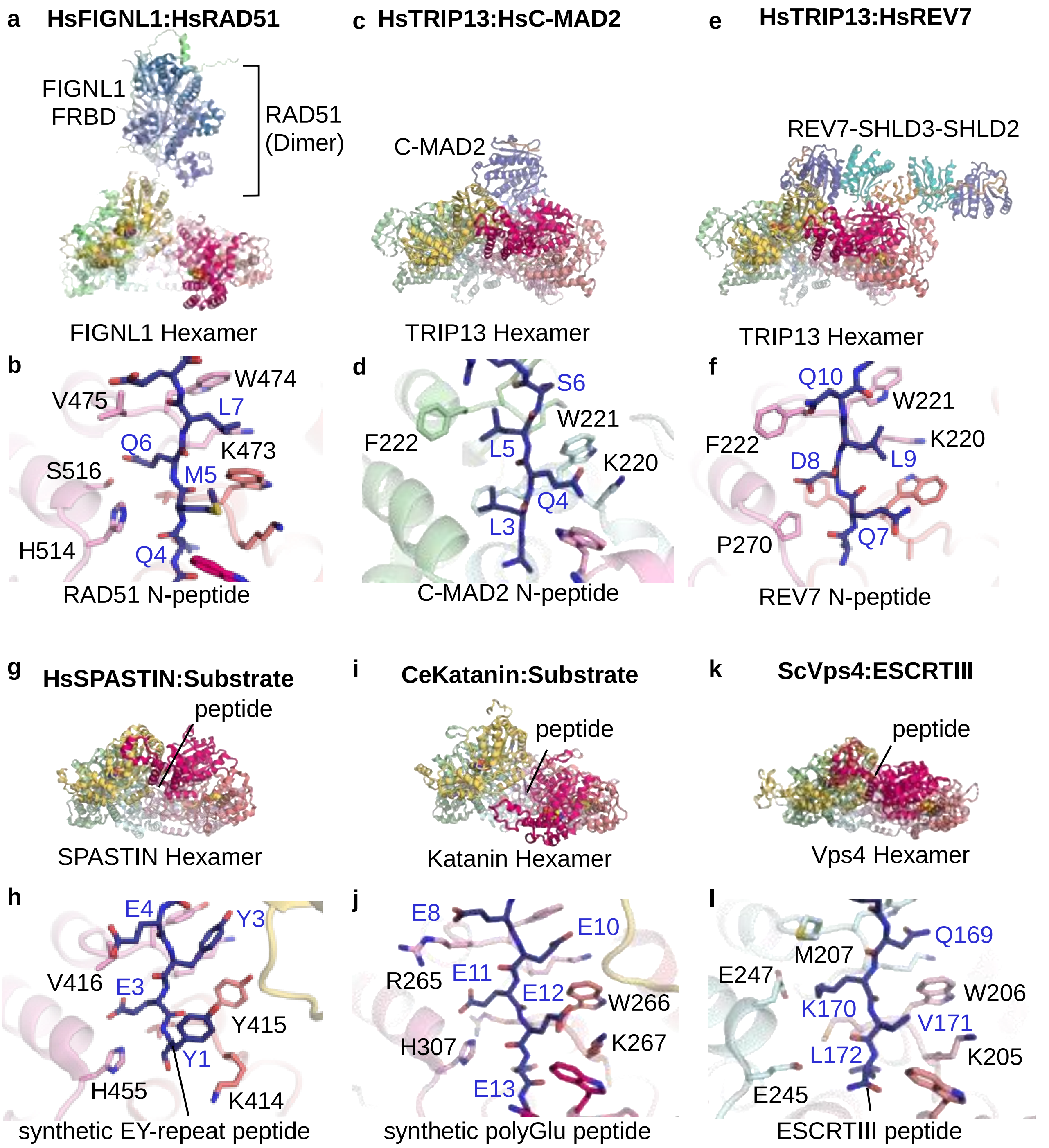

Fig. S15. Structural comparisons between FIGNL1 and peptide translocation AAA ATPases. (**A**) Composite atomic model of human FIGNL1-RAD51 using the cryoEM structure in combination with the Alphafold2 multimer predictions of FIGNL1-FRBD in complex with a RAD51 trimer. (**B**) Molecular details of the KWV-containing pore loop 1, and contributions from pore loop 2, interacting with the RAD51 N-terminus. (**C**) Structure of human TRIP13 bound to C-MAD2 (PDB 6F0X) alongside (**D**) the molecular details of the KWF-containing pore loop 1 interacting with the N-terminus of C-MAD2. (**E**) Structure of human TRIP13 bound to REV7 complex (PDB 7L9P) with (**F**) the molecular details of the pore loops interacting with REV7 substrate. (**G**) Structure of human SPASTIN

in complex with a synthetic peptide glutamate-tyrosine dipeptide repeat substrate (PDB 6PEN) with the molecular details of the KYV-containing pore loop 1 interacting with the substrate (**H**). (**I**) Structure of *C. elegans* Katanin in complex with a synthetic glutamate-rich peptide (PDB 6UGD) with the molecular details of the KWR-containing pore loop interacting with the substrate (**J**). (**K**) Structure of *S. cerevisiae* Vps4 in complex with an ESCRTIII-peptide substrate (PDB 6AP1) with the molecular details of the KWM-containing pore loop 1 interacting with the peptide (**L**). Structures were rendered in PyMol (Schrodinger). All structures are aligned with respect to the FIGNL1 hexamer and the subunits coloured by protomer (see Fig 3.) Substrates found threaded through the AAA+ hexamer pore are coloured dark blue.

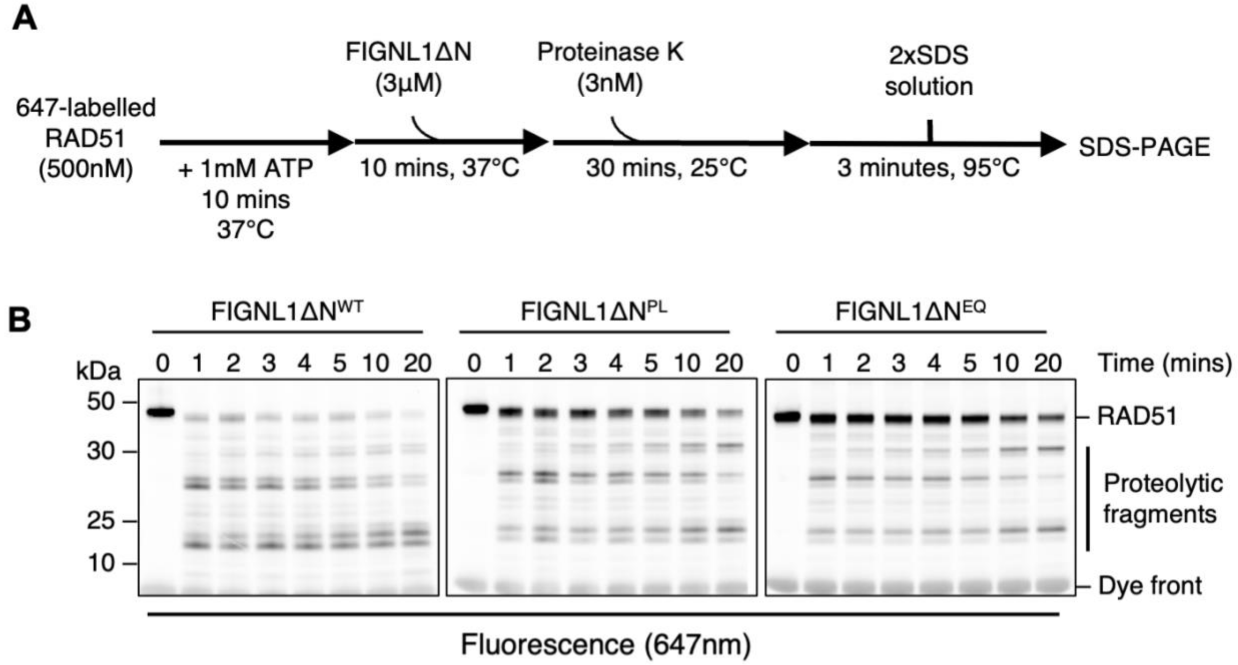

Fig. S16. Probing RAD51 conformation using limited proteolysis. (**A**) Limited proteolysis experiment scheme. (**B**) Proteinase K digestion of RAD51 in the presence of a 6-fold molar excess of either wild-type (WT), pore-loop mutant (PL), or Walker B (EQ) mutant FIGNL1ΔN at 25°C. Proteins and proteolytic fragments were resolved by 4-12% SDS-PAGE. RAD51-specific protein bands were visualised using a covalently attached fluorescent label (647nm). Each lane represents samples withdrawn at indicated time points. DNA was omitted in these experiments to remove conflating proteolytic patterns from filament to monomer transitions.

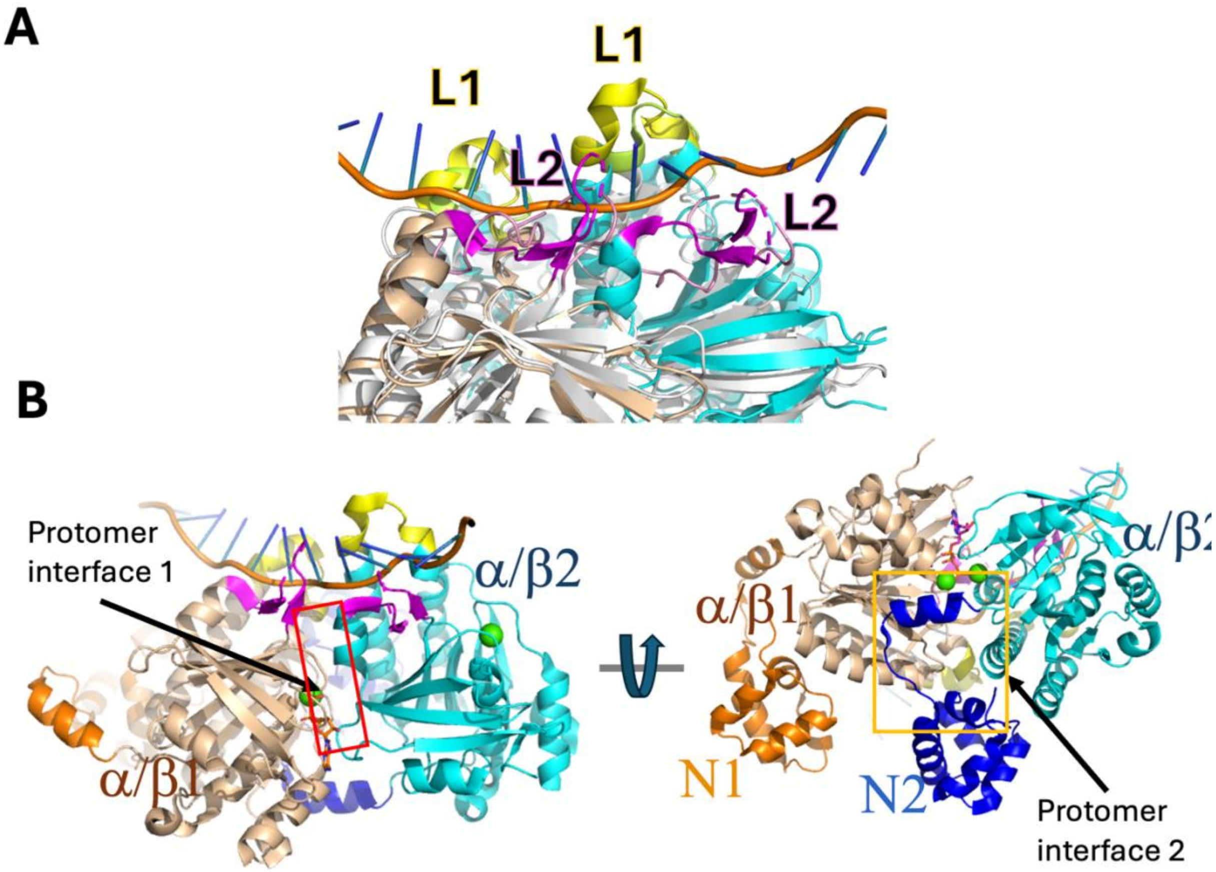

Fig. S17. Structural basis for stabilization of RAD51-DNA filaments. (A) RAD51 in Ca^{2+} -ATP bound ssDNA filaments (PDB code 8BQ2) uses two sites for DNA binding (L1- yellow and L2-magenta). (B) Orthogonal views showing the two interfaces between two adjacent RAD51 protomers in the filament. Interface 1 is formed between two α/β subdomains and interface 2 is bridged by N-terminal α-helical (N)-domain, purple) of protomer 2 (N2) bridges the two α/β subdomains ATP-bound RAD51: cyan and wheat, ADP-bound: grey. Ca^{2+}: green spheres, ATP is shown as sticks.

Table S1. **Cryo-EM data collection, refinement, and validation statistics**

	#FIGNL1ΔN^{E501Q}-RAD51 N-terminal peptide complex (PDB 8R64) (EMDB-18946)	FIGNL1ΔN^{E501Q}-RAD51
**Data collection and Processing (for each dataset)**		
Microscope	Titan KRIOS IV	Titan KRIOS IV
Voltage (keV)	300	300
Camera	Gatan K3	Gatan K3
Magnification	81000x	81000x
Pixel size at detector (Å/pixel)	1.1	1.1
Total electron exposure (e^{-}/Å^2)	50	50
Exposure rate (e-/pixel/sec)	8	8
Defocus range (μm)	-0.7 to -2.1	-0.7 to -2.1
Phase plate (if used)	N/A	N/A
- phase shift range (in degrees)	N/A	N/A
- number of images per phase plate position	N/A	N/A
Automation software (EPU, SerialEM or manual)	EPU	EPU
Tilt angle (if grid was tilted)	N/A	N/A
Energy filter slit width (if used)		
Micrographs collected (no.)	15406	15406
Micrographs used (no.)	15406	15406
Total extracted particles (no.)	2275651	369,634
**<u>For each reconstruction:</u>**		
Refined particles (no.)	275361	17582
Final particles (no.)	72562	17582
Point-group or helical symmetry parameters	N/A	N/A
Resolution (global, Å)		7.9
FSC 0.5 (unmasked/masked)	2.92	9.43
FSC 0.143 (unmasked/masked)	3.41/3.26	7.92
Resolution range (local, Å)	2.98/2.85	4.5-20
Resolution range due to anisotropy (Å)	2.9 to 5.6	N/A
	N/A	
Map sharpening *B* factor (Å^2) / (B factor Range)	-40	N/A
Map sharpening methods	CryoSPARC	N/A
**Model composition (for each model)**		
Protein	FIGNL1ΔN^{E501Q}	
Ligands	ATP, ADP, Mg^{2+}	
RNA/DNA	N/A	
**Model Refinement (for each model)**		
Refinement package	Phenix	
- real or reciprocal space	Real	
- resolution cutoff	5Å	
Model-Map scores		
-CC	0.81	
B factors (Å^2)		
Protein residues	69.2	
Ligands	60.1	
RNA/DNA	N/A	
R.m.s. deviations from ideal values		
Bond lengths (Å)	0.003	

Bond angles (°)	0.53
**Validation (for each model)**	
MolProbity score	1.33
CaBLAM outliers	0
Clashscore	6.98
Poor rotamers (%)	0
C-beta deviations	N/A
EMRinger score (if better than 4 Å resolution)	2.48
Ramachandran plot	
Favored (%)	98.68
Outliers (%)	0

Table S2.

Mutations found in FIGNL1 AAA+ domain and their pathogenicity scores from AlphaMissense (*19*), Cosmic database (*20*) or ClinVar(*21*).

Amino Acid	Position	Benign variants	Ambiguous variants	Pathogenic variants	Mean AM score	All benign variants	All ambiguous variants	All pathogenic variants	Mean AM score from all
K	382	4:E,Q,R,T	2:M,N		0.258	6:A,E,H,Q,R,T	7:G,I,L,M,N,S,V	6:C,D,F,P,W,Y	0.449
N	389	6:D,H,K,S,T,Y	1:I		0.201	13:A,C,D,G,H,K,L,M,Q,R,S,T,Y	4:E,F,I,V	2:P,W	0.324
G	405			4:A,E,R,V	0.923	None	None	19:A,C,D,E,F,H,I,K,L,M,N,P,Q,R,S,T,V,W,Y	0.947
L	430	1:I	1:V	2:F,S	0.612	1:I	2:M,V	16:A,C,D,E,F,G,H,K,N,P,Q,R,S,T,W,Y	0.839
R	431			6:G,K,M,S,T,W	0.878	None	None	19:A,C,D,E,F,G,H,I,K,L,M,N,P,Q,S,T,V,W,Y	0.883
G	432	1:A	2:R,V	1:E	0.455	3:A,C,S	3:R,T,V	13:D,E,F,H,I,K,L,M,N,P,Q,W,Y	0.59
P	434		1:A	5:H,L,R,S,T	0.707	None	1:A	18:C,D,E,F,G,H,I,K,L,M,N,Q,R,S,T,V,W,Y	0.867
F	440			6:C,I,L,S,V,Y	0.96	None	None	19:A,C,D,E,G,H,I,K,L,M,N,P,Q,R,S,T,V,W,Y	0.98
K	447			6:E,I,N,Q,R,T	0.979	None	None	19:A,C,D,E,F,G,H,I,L,M,N,P,Q,R,S,T,V,W,Y	0.992
L	449		1:V	4:I,P,Q,R	0.74	1:M	1:V	17:A,C,D,E,F,G,H,I,K,N,P,Q,R,S,T,W,Y	0.823
C	453			6:F,G,R,S,W,Y	0.9	None	None	19:A,D,E,F,G,H,I,K,L,M,N,P,Q,R,S,T,V,W,Y	0.944
G	459	3:A,E,R	1:V	1:W	0.365	10:A,C,D,E,H,K,N,Q,R,S	2:T,V	7:F,I,L,M,P,W,Y	0.433
S	464	2:C,G	2:N,T	2:I,R	0.504	4:A,C,G,L	2:N,T	13:D,E,F,H,I,K,M,P,Q,R,V,W,Y	0.663
S	466		2:A,T	4:C,F,P,Y	0.77	None	2:A,T	17:C,D,E,F,G,H,I,K,L,M,N,P,Q,R,V,W,Y	0.892
S	469	2:A,T	1:C	3:F,P,Y	0.616	2:A,T	2:C,L	15:D,E,F,G,H,I,K,M,N,P,Q,R,V,W,Y	0.785
K	473		1:R	5:E,I,N,Q,T	0.883	None	1:R	18:A,C,D,E,F,G,H,I,L,M,N,P,Q,S,T,V,W,Y	0.956
G	476			6:A,C,D,R,S,V	0.941	None	None	19:A,C,D,E,F,H,I,K,L,M,N,P,Q,R,S,T,V,W,Y	0.971
R	483		1:H	5:C,G,L,P,S	0.796	None	3:H,K,Q	16:A,C,D,E,F,G,I,L,M,N,P,S,T,V,W,Y	0.841
E	501			6:A,D,G,K,Q,V	0.997	None	None	19:A,C,D,F,G,H,I,K,L,M,N,P,Q,R,S,T,V,W,Y	0.998Q6PIW4
S	504		1:A	5:C,F,P,T,Y	0.803	None	1:A	18:C,D,E,F,G,H,I,K,L,M,N,P,Q,R,T,V,W,Y	0.893
E	515	1:D		5:A,G,K,Q,V	0.83	1:D	None	18:A,C,F,G,H,I,K,L,M,N,P,Q,R,S,T,V,W,Y	0.932
S	516	1:A	1:T	4:C,F,P,Y	0.653	1:A	2:G,T	16:C,D,E,F,H,I,K,L,M,N,P,Q,R,V,W,Y	0.809
K	521			6:E,I,N,Q,R,T	0.941	None	None	19:A,C,D,E,F,G,H,I,L,M,N,P,Q,R,S,T,V,W,Y	0.978
E	523			6:A,D,G,K,Q,V	0.948	None	None	19:A,C,D,F,G,H,I,K,L,M,N,P,Q,R,S,T,V,W,Y	0.977
A	531	3:S,T,V		3:E,G,P	0.534	3:S,T,V	3:I,L,M	13:C,D,E,F,G,H,K,N,P,Q,R,W,Y	0.708
D	537	2:E,N	2:A,G	3:H,V,Y	0.505	2:E,N	3:A,G,S	14:C,F,H,I,K,L,M,P,Q,R,T,V,W,Y	0.7
R	538	2:C,H	2:G,L	2:P,S	0.448	5:C,H,K,Q,W	8:E,G,I,L,M,T,V,Y	6:A,D,F,N,P,S	0.473
V	541		2:L,M	3:A,E,G	0.721	1:I	2:L,M	16:A,C,D,E,F,G,H,K,N,P,Q,R,S,T,W,Y	0.842
R	556			5:G,L,P,Q,W	0.834	None	None	19:A,C,D,E,F,G,H,I,K,L,M,N,P,Q,S,T,V,W,Y	0.899
S	571	4:A,L,P,T			0.069	19:A,C,D,E,F,G,H,I,K,L,M,N,P,Q,R,T,V,W,Y	None	None	0.13
E	592	5:A,D,G,K,Q	1:V		0.288	6:A,D,G,K,N,Q	4:R,S,T,V	9:C,F,H,I,L,M,P,W,Y	0.553
Q	595	5:E,H,K,L,R	1:P		0.139	18:A,C,D,E,F,G,H,I,K,L,M,N,R,S,T,V,W,Y	1:P	None	0.147
A	602	5:E,G,S,T,V		1:P	0.222	17:C,D,E,F,G,H,I,K,L,M,N,Q,R,S,T,V,Y	1:W	1:P	0.238
T	609	4:A,K,R,S	1:I	1:P	0.345	10:A,H,K,L,M,N,Q,R,S,V	4:F,G,I,Y	5:C,D,E,P,W	0.386
R	621		2:C,H	4:G,L,P,S	0.787	None	3:C,H,Q	16:A,D,E,F,G,I,K,L,M,N,P,S,T,V,W,Y	0.85
P	633	6:A,L,Q,R,S,T			0.121	13:A,E,G,H,I,K,L,M,Q,R,S,T,V	3:C,N,Y	3:D,F,W	0.296
Q	635	3:E,K,R	2:H,L	1:P	0.375	3:E,K,R	9:A,D,G,H,L,M,N,S,T	7:C,F,I,P,V,W,Y	0.552
Y	641	5:C,F,H,N,S	1:D		0.188	12:A,C,F,G,H,I,L,N,R,S,V,W	5:D,K,M,Q,T	2:E,P	0.314
R	649	5:G,I,K,S,T			0.177	15:A,C,E,G,H,I,K,L,M,Q,S,T,V,W,Y	3:D,F,N	1:P	0.236
R	652	1:Q		3:G,L,P	0.578	4:C,H,K,Q	1:W	14:A,D,E,F,G,I,L,M,N,P,S,T,V,Y	0.65
D	659			7:A,E,G,H,N,V,Y	0.795	None	None	19:A,C,E,F,G,H,I,K,L,M,N,P,Q,R,S,T,V,W,Y	0.887

(green)	Mutants from Cosmic database
(grey)	Variants of unknown significance from ClinVar
